# Effect of Nanoscale Zero-Valent Iron on Arsenic Bioaccessibility and Bioavailability in Soil

**DOI:** 10.3389/fchem.2022.964893

**Published:** 2022-07-22

**Authors:** Shuo Chen, Lei Han, Qiu Wang, Chenglang Liu, Yuzhen Liu, Jie Li

**Affiliations:** ^1^ College of Geography and Environment, Shandong Normal University, Jinan, China; ^2^ Jinan Environmental Research Institute (Jinan Yellow River Basin Ecological Protection Promotion Center), Jinan, China; ^3^ Jinan Ecological Environment Bureau Licheng Branch Bureau, Jinan, China

**Keywords:** arsenic, nanoscale zero-valent iron, sequence extraction, bioavailability, bioaccessibility

## Abstract

Hand-to-mouth activity is considered to be the main way for children to come into contact with contaminated soil, and bioavailability is an important factor affecting their health risk. To reduce soil As risk to humans by oral exposure, nanoscale zero-valent iron (nZVI) has been extensively studied for immobilizing As-contaminated soil, but its efficiency has not been investigated using *in vitro* assay and its influence on As-RBA. In this study, two contaminated soil samples (A and B) were amended with 1% and 2% (w/w) nZVI for 56 days to study its effect on As fraction by sequence extraction, As bioaccessibility by SBRC assay, and As relative bioavailability (RBA) by the mouse liver and kidney model. Based on the sequence extraction, the As associated with the E1 (exchangeable fraction) and C2 (carbonate fraction) fractions were decreased from 3.00% to 1.68% for soil A and from 21.6% to 7.86% for soil B after being treated with 2% nZVI for 56 days. When assessing As bioaccessibility in all soils treated with nZVI by SBRC assay, it was found that As bioaccessibility was significantly higher in the gastric phase (GP) and lower in the intestinal phase (IP) (*p* < 0.05), and the bioaccessible Fe concentration decreased significantly from the gastric to intestinal phase at the same time. Based on the mouse liver–kidney model, the As-RBA in soil A increased from 21.6% to 22.3% and 39.9%, but in soil B decreased from 73.0% to 55.3% and 68.9%, respectively. In addition, there was a significant difference between As bioaccessibility based on GP or IP of SBRC assay and As-RBA in two soils after being treated with nZVI for 56 days. To more accurately assess the effects of nZVI human arsenic exposure, As-RBA should be considered in concert with secondary evidence provided through fraction and bioaccessibility assessments. In addition, it is necessary to develop a suitable *in vitro* assay to predict As-RBA in nZVI-amended soils.

## GRAPHICAL ABSTRACT

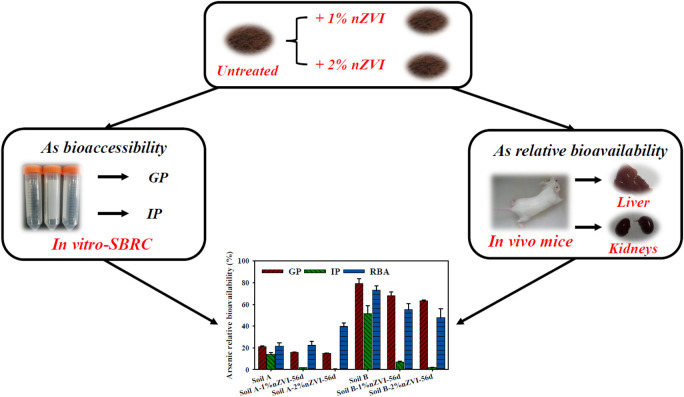

## Introduction

Arsenic (As) is toxic to humans and widely exists in the environment. Anthropogenic activities such as mining, smelting, and chemical manufacturing have elevated As concentrations in soil (Li et al., 2017). Long-term exposure to As not only causes cancer but also has negative effects on the development of the nervous system in children (Tokar et al., 2013; Manju et al., 2017). Soil exposure is an important way for human exposure to As, especially for children. It is estimated that the daily intake of soil by children was about 50–200 mg (Li et al., 2015b). After ingestion of contaminated soil, only part of As will be absorbed by organisms, that is, the bioavailable part (Juhasz et al., 2007). Thus, the main objective of many effective remediation strategies is to reduce the bioavailable fraction in soil.

At present, some efforts are focused on the stabilization of As to limit its mobility, bioavailability, and bioaccessibility using different soil amendments (Bolan et al., 2014). The development of nanomaterials specially designed for soil remediation has also attracted significant attention (Huang and Wang, 2016). Nanoscale zero-valent iron (nZVI) is one of the most widely nanomaterials used for treatments of metal (loid) contaminated soils (Fajardo et al., 2012). This material is characterized by high surface area to volume ratios and high surface energies (Wang and Zhang, 1997). Zero-valent iron has reducibility, which means that it can reduce its toxicity by changing the valency of As (Ramos et al., 2009). High surface energy can adsorb As and reduce their migration ability (Gupta et al., 2012; Wang et al., 2016). Many studies have discussed the effect of nZVI concentration on soil As pollution control (Fan et al., 2020; Li et al., 2021c). Although the benefits of this strategy are evident, governments must weigh the associated environmental and health risks. Many standardized ecotoxicity testing methods have been used to assess the effects of nZVI on environmental risks (Gil-Diaz et al., 2014; Fajardo et al., 2015). A significant effect of nZVI application on microbial structure has been recorded in the Pb-contaminated soil (Fajardo et al., 2015). The phytotoxicities of the soil samples were evaluated using a germination test with two plant species (*Hordeum vulgare L* and *Vicia sativa L*) (Gil-Diaz et al., 2014). Nevertheless, to fully assess the risks of nanomaterials better, more and more attention has been paid to human health risks.

The health risks of As in soil can be well assessed by *in vivo* and *in vitro* assays. The relative bioavailability (RBA), the fraction that is absorbed into the systemic circulation, obtained by animal models are more accurate than *in vitro* assays (Juhasz et al., 2014; Li et al., 2016b). Animal models including mice and swine have been used to confirm As-RBA in contaminated soil, but cost and time consideration limit their use on a large scale (Juhasz et al., 2009; Li et al., 2015b). However, the *in vitro* assays have the advantages of being low-cost and having a short experimental period. As a result, *in vitro* gastrointestinal assays have been developed as surrogate assays for estimating As-RBA (Juhasz et al., 2009; Li et al., 2015b; Li et al., 2019). The As bioaccessibility refers to the fraction of As that is soluble in gastrointestinal fluid and therefore potentially available for absorption into systemic circulation (Li et al., 2021b). Juhasz et al. (2014) demonstrated the capacity of the SBRC gastric phase (GP) to accurately predict As-RBA in contaminated soil. Li H.-B. et al. (2015) measured As bioaccessibility using four *in vitro* assays and showed that IVG has the potential to measure As bioavailability in contaminated soils from China though UBM and SBRC assays were also suitable.

Most of the previous studies *in vivo* and *in vitro* correlations were used to test the ability of *in vitro* assays to predict As-RBA in contaminated soil (Li et al., 2015a; Li et al., 2015b; Li J. et al., 2016b). However, there is a lack of comparison of As-RBA and As bioaccessibility in amended soils. Li et al. (2017) reported four *in vitro* assays were not suitable to predict As-RBA in phosphate-amended soils (Li et al., 2017). Similarly, *in vitro* assays may not be able to accurately predict Pb-RBA when nZVI was used by comparing the *in vivo* and *in vitro* results (Mele et al., 2015). Nanoscale zero-valent iron (nZVI), as one wide amendment of As-contaminated soil, has the properties of nanometer structure and iron. The suitability of *in vitro* assays to assess the influence of nZVI amendment on As-RBA is unclear. Therefore, the comparison of As-RBA and As bioaccessibility in nZVI-amended soils need further research.

The objective of this study was to study the influence of nZVI on As bioaccessibility and As-RBA in soils. Arsenic bioaccessibility was measured by the use of the SBRC method, while As-RBA was estimated by a mice model with kidneys and liver as endpoints. The specific objectives of this study were to: (1) determine the effect of nZVI on As fractions in two soil samples by the use of sequential extraction; (2) measure As bioaccessibility by the use of SBRC assay before and after different nZVI dosages treated the soils; (3) measure As-RBA by the mice model before and after different nZVI dosages treated the soils; (4) assess the ability of SBRC assay to predict As-RBA in nZVI-amended soils.

## Materials and Method

### Contaminated Soils

Two contaminated soil samples (soil A and soil B) were collected from the Hunan and Henan provinces of China. They were air-dried at room temperature and sieved to a < 250 μm size fraction and thoroughly mixed in a plastic bag by shaking end-over-end. This particle size is easy for children to be ingested through the hand-mouth pathway (Li et al., 2016b).

The air-dried samples A and B were digested in duplicate by the USEPA method 3050B. Briefly, the soil samples were weighed in polypropylene digest tubes, and then digested completely with 1:1 HNO_3_ and 30% H_2_O_2_ at 95°C on a graphite digester. Previous studies showed that mineral elements (for example, Fe, Zn, Mn, Ca, Cu, etc.) can reduce the bioaccessibility and RBA of heavy metals in the soil (Zhao et al., 2017). For instance, Fe is the main factor affecting bioavailability in acidic soil, while Ca is more important in alkaline soil. Total concentrations of As and mineral elements in soil digestion solution were determined by inductively coupled plasma-mass spectrometry (ICP-MS, iCAP RQ, Thermo Scientific, United States) and inductively coupled plasma optical emission spectrometry (ICP-OES, Optima 5300DV, PerkinElmer, United States). After shaking for 2 h, the pH value of soil in water extract (solid-liquid ratio was 1:5) was determined. After the carbonate was removed by HCl, the content of organic matter (OM) was determined by a resistance furnace (SX2-4-13G, ELITE-LAB instrument, China). Soil mineralogy was determined on finely ground samples by X-ray diffraction (XRD, Ultima Rigaku, Japan). Continuous scans from 5 to 85° at 2θ were collected with a step size of 0.01° and a count time of 0.2s per step. Samples for XRD were crushed, dried and sieved to 250 μM.

### Soil Treatments

Two soil samples treatments were prepared by combining 30 g of soil with nZVI. Soil A and B served as control samples. All amendments were prepared in triplicate on 1% and 2% (w/w) nZVI, and dosages were adjusted considering practicalities and economic costs based on previous studies (Sneath et al., 2013). Following the addition of nZVI, treated and controlled soils were thoroughly mixed in sealed bags by shaking with hands. All soils were stored at 25C and maintained at a 60% water holding capacity by daily weight adjustment with Milli-Q water for 56 days. During this period, all samples were mixed every 3 days and collected at 1 day, 2 days, 3 days, 7 days, 14 days, 28 days, and 56 days.

### Sequential Extraction of Soils

In this study, As in the 6 samples were divided into 5 fractions *via* sequential extraction (Tessier et al., 1979). These 5 fractions include: E1, exchangeable fraction; C2, carbonate fraction; F3, Fe/Mn oxide fraction; O4, organic fraction; and R5, residual fraction. After each extraction, the soil residues were washed 3 times using Milli-Q water to remove As released from the previous steps. Different from the traditional method, we use the USEPA3050B digestion method when dealing with the R5 fraction.

For QA/QC, certified reference materials (GSS-5) and reagent blank were used to check the accuracy and precision of the analytical data for speciation concentration. The total As in GSS-5 was 412 ± 24 mg kg^−1^. After sequential extraction, the amount of five fraction of As was 423 ± 5.42 mg kg^−1^.

Due to the addition of nZVI to the soil, the speciation distribution of Fe in the samples before and after aging was worthy of attention. The dithionite–citrate bicarbonate (DCB) extraction method can effectively extract free iron oxide from soil samples, while the oxalate-ammonium oxalate extraction method can be used to extract amorphous iron oxide (Jiang et al., 2016; Nawaz et al., 2016). The content of Fe in the samples was analyzed by ultraviolet-visible spectrometer (N4S, INESA analytical instrument, China).

### Arsenic Bioaccessibility *via* SBRC Assay

In this study, the SBRC assay was used to measure As bioaccessibility in soils, with 0.2 g of soil sample being extracted in the gastric phase using 20 ml of gastric fluid consisting of 30.03 g L^−1^ glycine in 1 L of Milli-Q water, using HCl to adjust the pH of solution to 1.5. The samples were shaken at 37°C and 150 rpm for 1 h. During this period, the solution’s pH was monitored and adjusted to 1.5 using concentrated HCl. The sample was centrifuged at 4,000 rpm for 10 min and then 10% of the solution volume was collected (Li et al., 2015a; Li et al., 2021b).

For the intestinal phase extraction, the extraction solution was modified by adjusting the pH to 7.0 with NaOH and addition of 1.75 g L^−1^ bile and 0.5 g L^−1^ pancreatin. 10 ml of solution was collected and filtered (0.45 μM) after 4 h extraction. All samples were stored at 4°C before analysis by ICP-MS. As bioaccessibility was calculated by dividing the extractable arsenic in solution by the total arsenic in soil samples.

For QA/QC, a soil standard reference material (SRM NIST2711a, National Institute of Standards and Technology) was included. Bioaccessible As in the SRM in GP and IP was 59.2 ± 0.27 and 45.9 ± 0.61 mg kg^−1^, consistent with 60.7 ± 1.63 and 46.8 ± 0.60 mg kg^−1^ was reported by Li et al. (2015b) using SBRC assay.

### Determination of As-RBA by the Mouse Model

In this study, *in vivo* bioassays were conducted using female Balb/c mice with a body weight (bw) of 18–20 g. The mice needed to be acclimatized for 7 days. During this period, mice had free access to Milli-Q water containing <5 ng L^−1^ As and a rodent diet obtained from the Pengyue Experimental Animal Breeding Farm (Jinan, China) containing 0.43 ± 0.02 mg of As kg^−1^, and received a 12/12 light/dark photocycle at 20–22°C. Then the mice were fasted overnight and placed separately in the polyethylene cages the next day, one in each cage, and three in a group. Animal care was compliant with the Guide for the Care and Use of Laboratory Animals at Shandong Normal University.

The steady state multiple dosing approach *via* feed was utilized. The treatment group included an untreated control group and soil-modified diet groups. For the next 10 days, each mouse was free to drink Milli-Q water and received 5 g of feed every day. The soil exposure feed was prepared with the ratio of soil to feed at 1:30 in the basic diet of mice. The range of As concentration in amended diet was 3.92–4.32 mg kg^−1^, and the dose level was 0.47–0.87 mg As kg^−1^ bw d^−1^. At the same time, sodium arsenate was used as the standard substance to prepare diets with an As concentration of 1–100 mg kg^−1^ and a dose level of 0.11–7.66 mg As kg^−1^ bw d^−1^. Ten days later, the mice were fasted overnight and weighted, and then sacrificed to collect liver and kidney samples. The samples were stored at −80°C before freeze-drying and were digested with concentrated HNO_3_ and 30% H_2_O_2_ according to the USEPA method 3050B (Zhao et al., 2018). Detailed information about soil-amended diet preparation is shown in Supplementary Table S1 in the Supporting Information (SI).

Initially, a linear dose response curve (DRC) of As concentration in kidneys and livers following sodium arsenate exposure was established (Supplementary Figure S1). The arsenic-RBA content in soils was then calculated as the ratio of dose normalized As concentration in kidney and liver after soil exposure to the slope of the corresponding DRC for sodium arsenate.

$$\mathrm{As}-\mathrm{RBA}\left(\%\right)=\frac{\mathrm{target organs As after soil exposure}}{\;\mathrm{As dose level via soil}\times \mathrm{slope of DR}C_{\mathrm{sodium arsenate}}}\times 100\%.$$

For QA/QC, SRM NIST2710a was incorporated into a basic mouse diet to achieve an As concentration of 5 mg As kg^−1^, which was similar to the soil dosage. The relative bioavailablity of As in SRM NIST 2710a was 43.5 ± 13.8%, in agreement with 33.2% reported by Li H.-B. et al. (2021a).

### Statistical Analysis

The results are presented as average values ± standard deviation. The figures were done by SigmaPlot 14.0. The data were analyzed by SPSS software (version 26.0, Chicago, United States). The means were tested for significance at *p* = 0.05. The data obtained by XRD were analyzed by MDI Jade 6, and the phase was considered to exist when FOM < 10.

## Results and Discussion

### Effects of Nanoscale Zero-Valent Iron Amendment on Soil Properties and Arsenic Fraction

**FIGURE 1:**
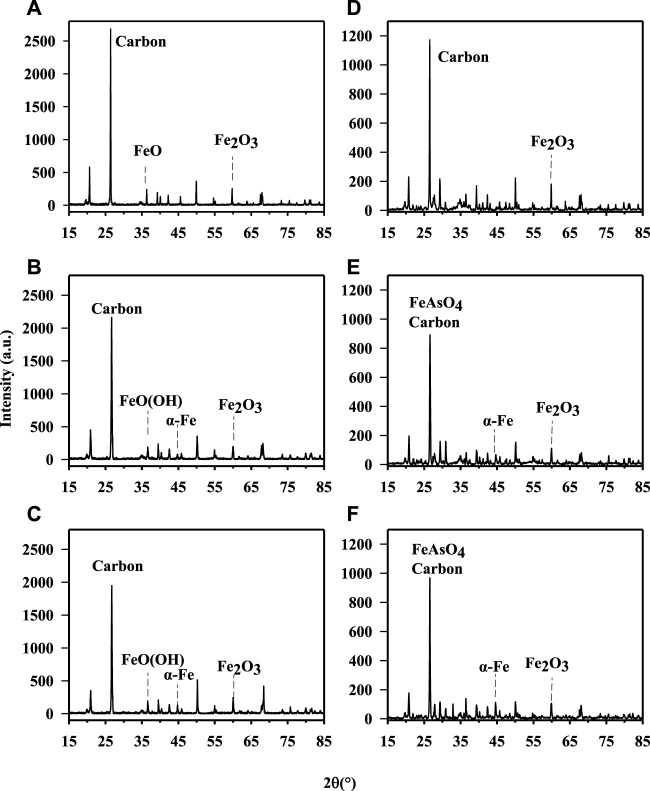
X-ray diagram of untreated and treated soils with different nZVI dosages. **(A)** soil A. **(B)** soil A treated with 1% nZVI for 56 days. **(C)** the soil A treated with 2% nZVI for 56 days. **(D)** soil B. **(E)**soil B treated with 1% nZVI for 56 days. **(F)** soil B treated with 2% nZVI for 56 days.

The physical and chemical properties of two As contaminated soil samples from different sources amendment by different amounts of nZVI are listed in Table 1, including the total concentrations of key elements, soil pH and OM. The total As concentration of soil A from Hunan was 129 ± 11.8 mg kg^−1^, and that of Henan soil was 121 ± 9.58 mg kg^−1^. The contents of Ca, Mn, Zn, Al, and Cu in soil A and B were analyzed and compared, and it was found that there were significant differences in the contents of Mn and Ca, respectively (*p* < 0.05), while there were no significant differences in the contents of Zn, Al and Cu in the two samples (*p* > 0.05). The soil A from Hunan was acidic and the soil B from Henan was alkaline. The XRD analysis confirmed the presence of zero-valent iron and iron oxides, mainly represented by ferric oxide (Fe_2_O_3_) (Figure 1). After the treatment with nZVI during the 56 days, the difference in pH across all treatments was <1 pH unit (Table 1). The OM of soil samples decreased from 4.99 to 6.45% to 3.67–6.01% after nZVI treatment. The result was consistent with the XRD data, with the increase of nZVI, the carbon content decreases obviously (Figure 1). This is due to the reducibility of nZVI, and the addition of nZVI to the soil is easy to react with oxide minerals and organic matter in the soil, resulting in a decrease in the content of soil organic matter (El-Temsah et al., 2016).

**TABLE 1:** Selected properties of 2 contaminated soils and 4 nZVI treatment soils after 56 days (< 250 μM particle size).

Sources	Sample ID	Treatment	pH	OM (%)	Total Fe (g kg-1)	Total Mn (g kg^−1^)	Total Ca (g kg^−1^)	Total Zn (g kg^−1^)	Total Al (g kg^−1^)	Total Cu (g kg^−1^)	Total As (mg kg^−1^)
Hunan	Soil A	untreatment	6.1	6.45	22.7 ± 4.49	0.19 ± 0.05	1.91 ± 1.26	1.28 ± 0.47	3.00 ± 0.14	0.08 ± 0.03	129 ± 11.8
		soil A-1% nZVI	5.72	6.01	32.6 ± 3.25	0.17 ± 0.02	2.01 ± 0.78	1.09 ± 0.62	3.11 ± 0.03	0.06 ± 0.01	121 ± 12.6
		soil A-2% nZVI	5.8	5.73	41.7 ± 0.62	0.23 ± 0.04	2.06 ± 0.59	1.15 ± 0.29	3.02 ± 0.28	0.08 ± 0.01	127 ± 9.81
Henan	Soil B	untreatment	8.02	4.99	22.4 ± 1.68	0.49 ± 0.04	14.8 ± 0.21	1.13 ± 0.05	3.37 ± 0.03	0.08 ± 0.01	121 ± 9.58
		Soil B-1% nZVI	8.07	5.06	31.8 ± 0.94	0.51 ± 0.01	13.5 ± 1.06	1.10 ± 0.02	3.29 ± 0.10	0.09 ± 0.01	119 ± 5.38
		Soil B-2% nZVI	8.08	3.67	43.1 ± 1.14	0.47 ± 0.01	14.7 ± 0.03	1.09 ± 0.08	3.36 ± 0.01	0.08 ± 0.02	124 ± 6.97

After the addition of nZVI, the speciation of Fe in the soil deserves attention. First of all, we measured the total Fe in the soil samples after adding nZVI. The content of total Fe in soil samples increased significantly from 22.4–22.7 to 31.8–43.1 g kg^−1^. Then, we measured the concentrations of free Fe oxide (Dithionite–citrate bicarbonate) and amorphous Fe oxide (oxalate-ammonium oxalate) in As-contaminated soils before and after nZVI treatment, respectively (Supplementary Table S2). Both free Fe oxide (from 8.89 to 14.3 to 17.9–35.8 mg kg^−1^) and amorphous Fe oxide (from 2.65 to 8.00 to 8.21–30.4 mg kg^−1^) concentrations increased significantly.

To evaluate the effect of nZVI on As fraction in two soils with different properties, the Tessier sequential extraction method was used to extract As from soil. Figure 3 showed the changes of the As fractions in the soil samples obtained using the sequential extraction procedure before and after treatment with nZVI during 56 days. For the untreated soil (A and B), the majority of As was associated with the R5 fraction (68.1% and 60.5%), which was not available for soil organisms. However, there was still a high percentage As in the most available and potentially available soil fractions. The treatments with 1% and 2% nZVI induced a significant decrease in the As associated with the E1 and C2 fractions, and the lowest percentage was found at 2% nZVI (from 3.00% to 1.68% for soil A and from 21.6% to 7.86% for soil B). Similar result was reported by Gil-Diaz et al. (2014). No significant differences were found between 7days and 56 days of contact time between two soil samples and nZVI. Thus, the As-nZVI interaction resulted in stable at least for 56 days. The application of nZVI induced a reduction of As in E1 and C2 near 0.15%–1.72% and 5.67%–14.6% for soil A and B, respectively. In general, the reduction of As would be explained because the mechanism for As immobilisation with iron is the absorption of As onto the reactive surfaces of Fe. In this study, the arsenate can coprecipitate with Fe^3+^ as FeAsO4 which have low solubility was detected in soil B through XRD analysis (Kumpiene et al., 2006). However, no peaks of crystalline Fe-As compounds were detected in XRD analysis, most likely due to low concentrations of these newly formed mineral phases in soil A. In the F3 fraction, the application of 1% and 2% nZVI did not significantly change As fraction of two soil samples. Regarding the O4 fraction, a significant decrease (∼68% and ∼57%) were observed between the control soil samples and the soil treated with 1% and 2% nZVI for 56 days, in agreement with soil organic matter. Other authors have also obtained a similar decrease in O4 after treatment with 1% and 10% nZVI for 3 days and 3 months (Gil-Diaz et al., 2014). Furthermore, the As in R5 fraction which was in immobilised As increased 1.88%–14.9% after treatment with nZVI. Similar results were obtained with a significant increase in immobilized As in soil treated with 10% nZVI (Gil-Diaz et al., 2014). In summary, the results showed that a significant reduction of As in the more available fractions for soil samples treated with nZVI.

### Effects of Nanoscale Zero-Valent Iron Amendment on Arsenic Bioaccessibility

**FIGURE 2:**
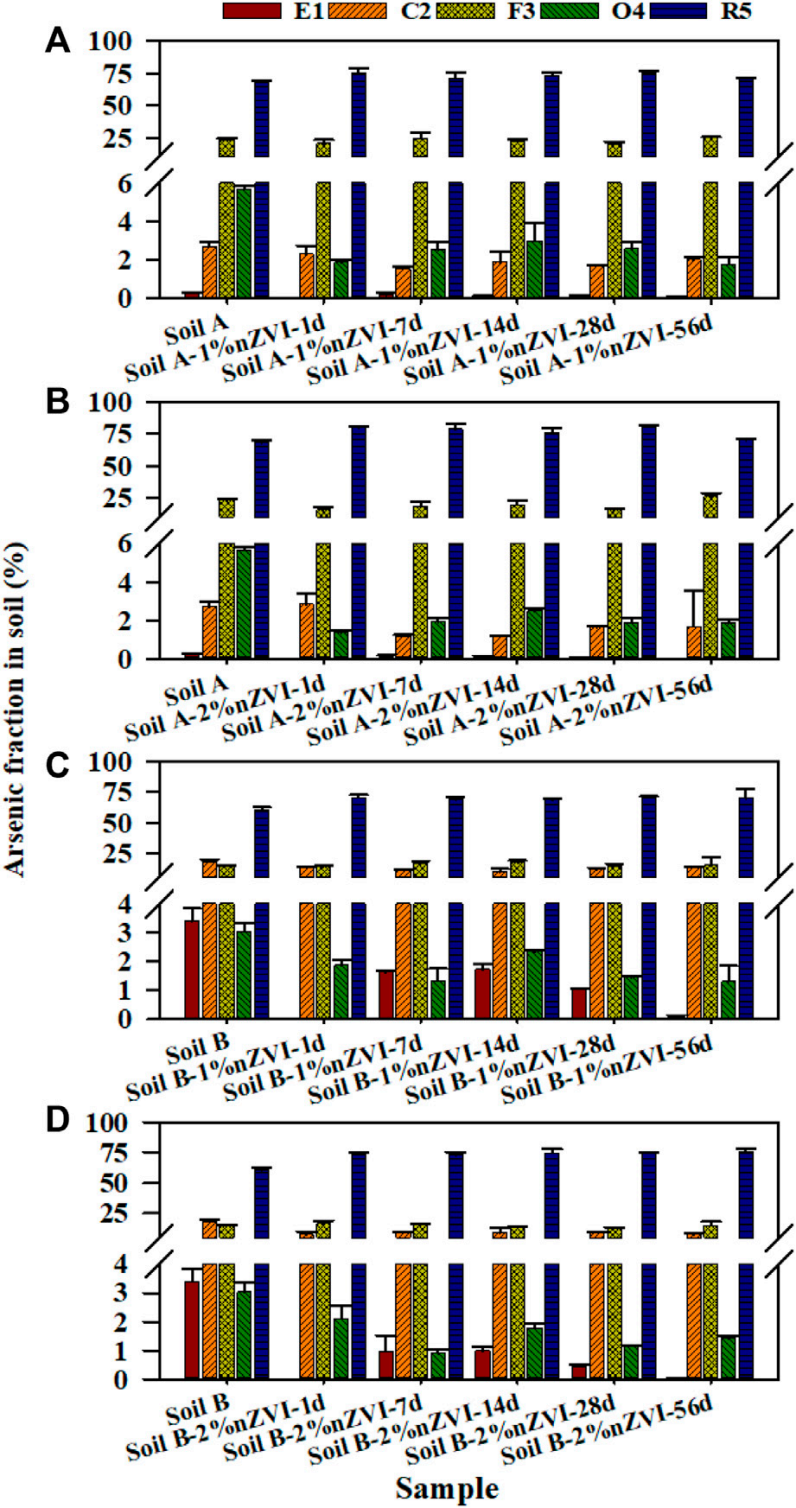
Arsenic distribution among 5 fractions for **(A)** Soil A-1% nZVI treatment, **(B)** Soil A-2% nZVI treatment, **(C)** Soil B-1% treatment and **(D)** Soil B-2% nZVI treatment following the Tessier method. Error bars represent the standard deviation of duplicate analyses.

**FIGURE 3:**
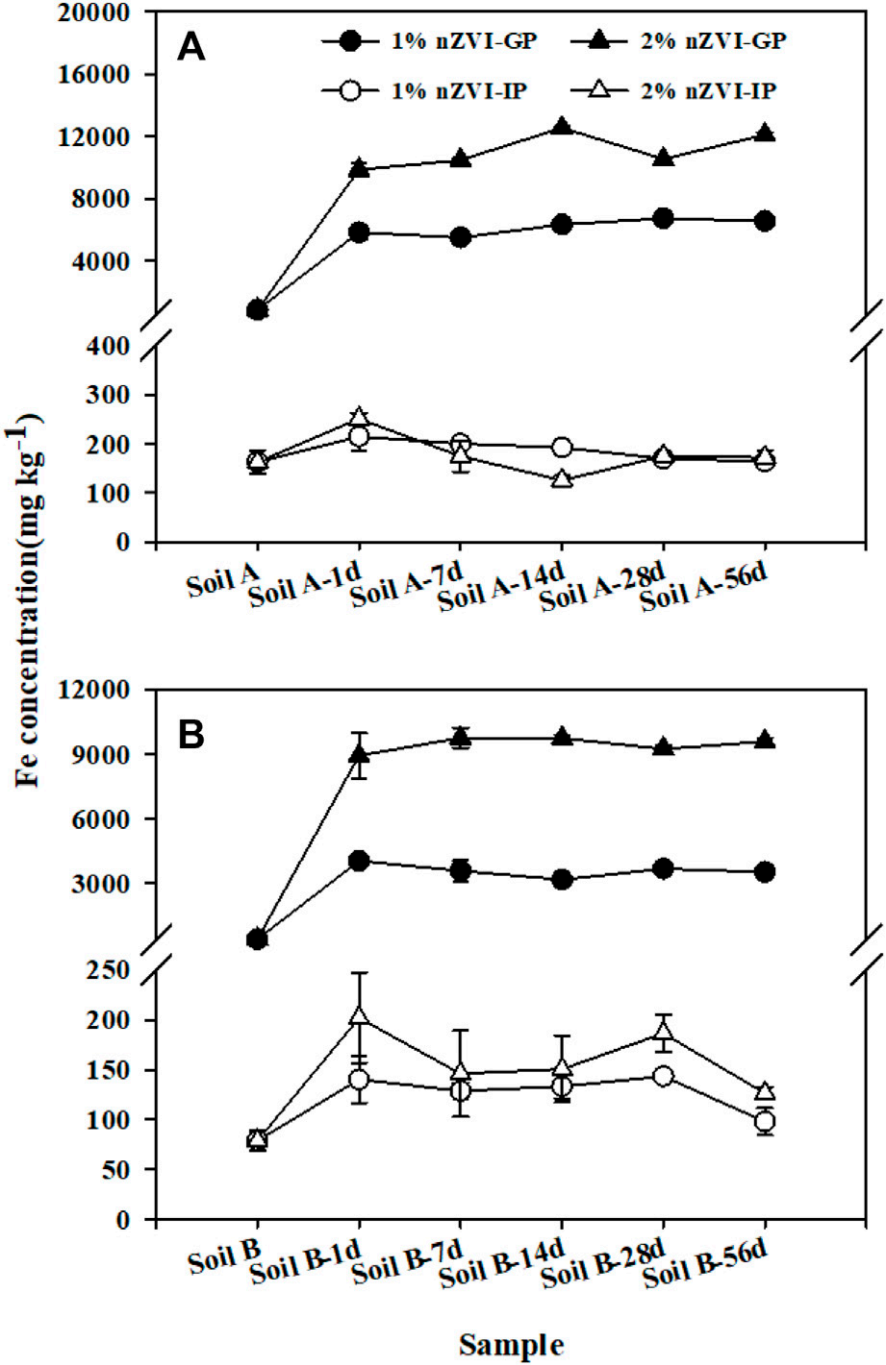
Fe concentration extracted from nZVI untreated and treated soil A **(A)** and B **(B)** during the gastric phase (GP) and intestinal phase (IP) of the SBRC assay. Data are expressed as mean and standard deviation of triplicates.

The bioaccessibility of As in soil A and B was 13.9 ± 1.87–21.2 ± 0.39% and 51.6 ± 7.34–79.2 ± 4.57% in gastric and intestinal phases, respectively, illustrating that the pH of the GP and IP play a lesser role in controlling As bioaccessibility (Figure 3). The differences in As bioaccessibility among two soils can be explained by their different As fractionations in soils. For soil A, only∼3.00% As was associated with available forms including E1 and C2, while about 21.5% in soil B (Figure 2). Except As fraction, the Ca in soil also played an imported role in controlling As bioaccessibility. Compared to soil A, soil B as alkaline soil has higher Ca concentration. Meunier et al. (2010) reported that the highest As bioaccessibility (up to 49%) was associated with the presence of calcium-iron arsenate in alkaline soils with high Ca concentration. For all the soil samples treated with nZVI, As bioaccessibility was generally higher for the gastric phase compared to the intestinal phase. A similar decrease in As bioaccessibility from the gastric to the intestinal phase has been observed by other researchers (Juhasz et al., 2009; Li et al., 2015b). From the gastric phase to the intestinal phase, the increase in pH resulting from modification of the intestinal phase conditions resulted in a change in Fe concentration. Figure 3 illustrated that the bioaccessible Fe concentration decreased significantly from the gastric to intestinal phase due to dissolved Fe from gastric phase dissolution becomes oversaturated and hydrolyzed Fe species precipitate as amorphous Fe structures. Due to the addition of nZVI, more dissolved As can be resorbed to the amorphous Fe by surface complexation or ligand exchange to the surface hydroxyl functional groups, thereby greatly reducing As bioaccessibility in the intestinal phase compared to that of soil A and B. Pearson’s correlation was established between Fe concentration in the extract and As bioaccessibility, and a significant negative correlation (R^2^ = 0.60–0.72) between Fe concentration and As bioaccessibility in the intestinal phase was observed (Supplementary Table S3).

**FIGURE 4:**
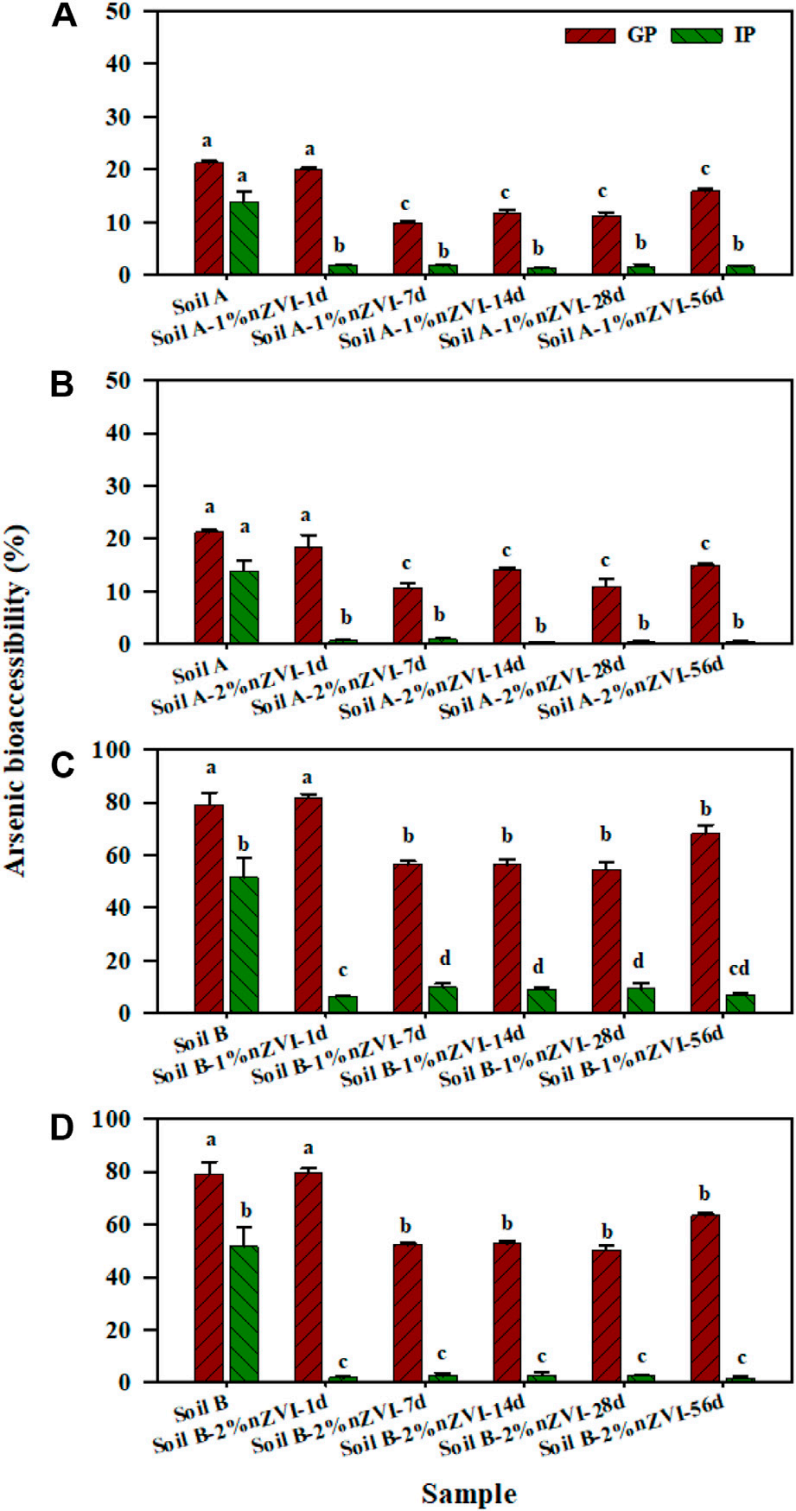
Arsenic bioaccessibility in nZVI untreated and treated soil A **(A,B)** and B **(C,D)** samples measured by the gastric phase (GP) and intestinal phase (IP) of the SBRC. Data are expressed as mean and standard deviation of triplicates. Same letters above error bars demonstrate there was no significant difference between As bioaccessibility in GP or IP.

To better compare the effect of nZVI on As bioaccessibiity, the treatment effect ratio (TER) was used, that is, the As bioaccessibility in the treated soil divided by the bioaccessibility in the untreated soil. In this study, no significant change of As bioaccessibility (TER∼1) was observed in the gastric phase. The XRD showed that Fe or As-Fe mineral (such as, FeO(OH), FeAsO_4_) was formed in treated soil samples with nZVI (Figure 1). However, the stability of formed As-Fe minerals was influenced by solution pH as they may be soluble at low pH (1.5) in SBRC gastric phase. Different from the gastric phase, As bioaccessibility of nZVI-treated samples decreased from 13.9–51.5% to 0.26–11.4% (TER ranged from 0.03 to 0.19) in the intestinal phase, although the reduction varied depending on the dosage of nZVI treatment used (Figure 4). This result was consistent with previous research with TER 0.63 *via* treating with 1% nZVI after 2 months (Mele et al., 2015)**.** These reductions in As bioaccessibility may be attributed to the increase in Fe mineral in the soils as these control the available of As.

### Effects of Nanoscale Zero-Valent Iron Amendment on Arsenic Relative Bioavailability

Although the SBRC method to determine the effect of nZVI on the As bioaccessibility has been carried out in the early stage, the current research on the effect of nZVI on As-RBA is still lacking. Based on the special structure of nZVI itself, it is extremely necessary to carry out the *in vivo* experiment. This study measured As-RBA in all treatment soils with an As concentration of 3.92–4.32 mg As kg^−1^ using an *in vivo* mouse model where the mice were exposed to soil or soil-nZVI amended diet for 10 days with As in the liver and kidneys being utilized as the biomarker (Supplementary Table S1).

Initially, the dose response of As accumulation in mouse kidneys and livers was established using a range of sodium arsenate (Na_2_HAsO_4_) concentrations (0–100 mg kg^−1^) in amended mouse diets (Supplementary Figure S1). Though As accumulation in both the liver and kidney showed strong linear response to administered As levels (R^2^ = 0.98 in liver, R^2^ = 0.95 in kidneys), As concentrations differed significantly in the tissues. Based on this, liver plus kidneys were used to determine As-RBA in soils. As the concentration in mouse liver and kidneys strongly and linearly correlated with dosage levels following sodium arsenate ingestion (R^2^ = 0.98).

**FIGURE 5:**
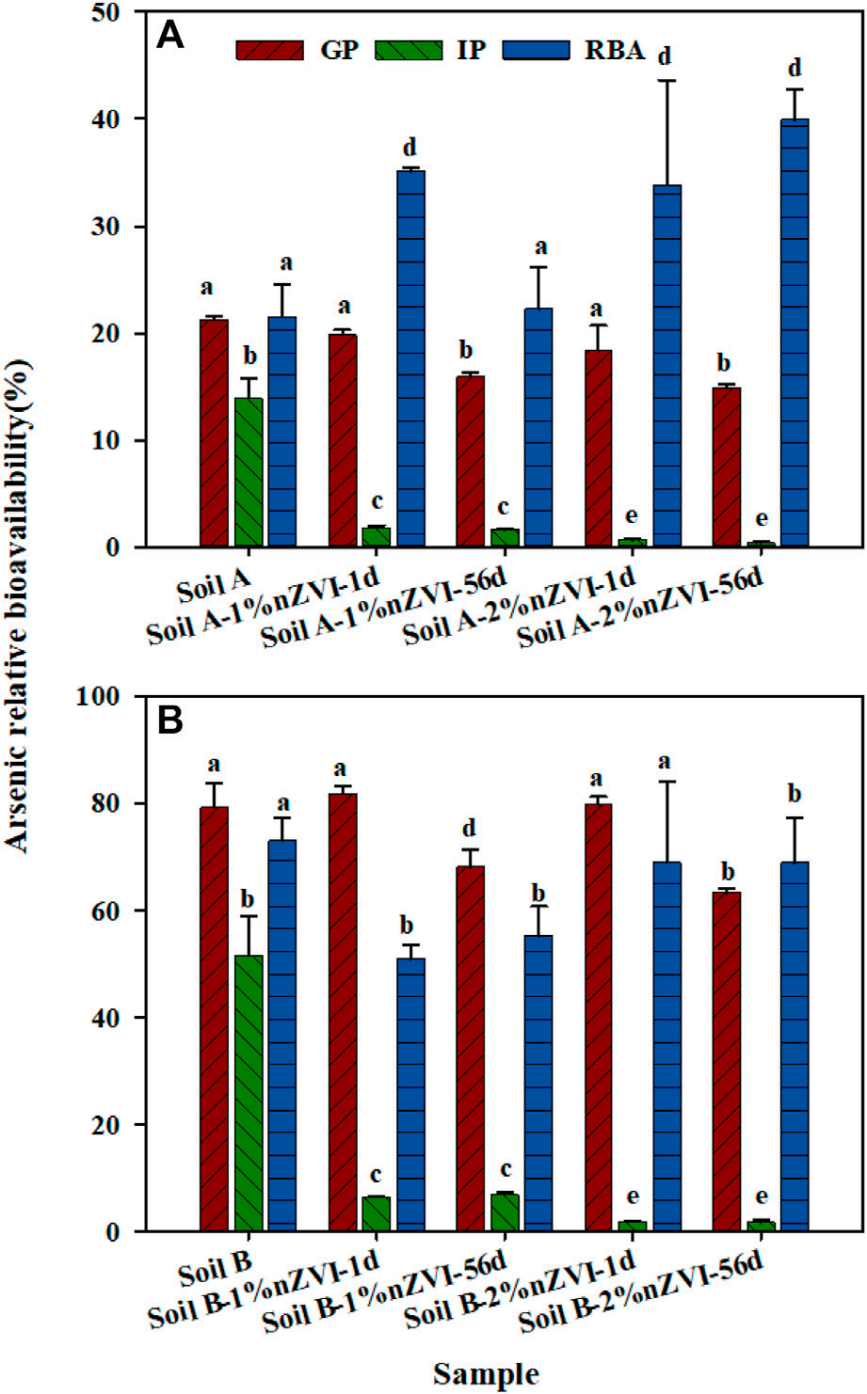
Comparison between As relative bioavailability (RBA) and bioaccessibility measured by the GP and IP of SBRC for **(A)** soil A, and **(B)** soil (B). Data are expressed as mean and standard deviation of triplicates. Same letters above error bars demonstrate there was no significant difference between bioaccessibility and relative bioavailability for the same sample.

On the basis of the linear response, all treatments soil-amended diets (3.92–4.32 mg As kg^−1^) were supplied to mice for 10 days to quantify As accumulation in livers and kidneys to measure As-RBA in all soil samples (Figure 5). The mice were fed with basic food as a control group, and the content of As in the liver and kidney was 18.4–44.2 μg kg^−1^, averaging 33.7 μg kg^−1^. After feeding the food mixed with untreated soil A and B for 10 days, the content of As in liver and kidneys increased to 118–731 μg kg^−1^, averaging 250 μg kg^−1^. Though the total As in soil A and B was similar, the As-RBA of soil A and B were varied from 21.5 to 73.0%. The difference was consisted with the As bioaccessibility measured by SBRC which may be caused by the available As fraction in two soil samples. Compared to the untreated soil A, the soil A treated with 1% nZVI had similar As-RBA, while As-RBA of soil A treated with 2% nZVI increased to 39.9% for 56 days. The results showed that the higher nZVI dosage played a double-sided role in As toxicity, reducing E1+C2 fraction (from 3.0% to 1.68%) and improving As-RBA. It’s reported that nZVI was easier to contact and bind with cells because of its surface charge (Wang et al., 2006). When the nZVI adsorbed with As entered into the cell and be transported to the target organs such as the liver, this process would increase As accumulation in mice and lead to an increase the As-RBA (Wang et al., 2010). Different from soil A, the As-RBA of soil B treated with 1% nZVI and 2% nZVI was decreased from 73.0% to 55.3%, and 68.9%, respectively. Though nZVI adsorbed As was easier to accumulate in the target organs, the soil B treated with 1% and 2% nZVI led to the E1+C2 As fraction significantly reduced from 21.6% to 13.5% and 7.86%. Based on the aforementioned results, we can conclude that the effect of nZVI on the As-RBA depends on the nZVI dosage and soil properties, especially the As fraction in soil samples.

### Comparison Effects of Nanoscale Zero-Valent Iron Amendment on As-RBA and Bioaccessibility

The bioaccessibility and bioavailability assays were performed to accurately evaluate the potential human exposure to metal (loids) in contaminated sites and consequently to discover possible remediation strategies. The addition of nZVI resulted in a decrease in As bioaccessibility for the two soil samples, which showed a great practical value. Previous studies have illustrated the capacity of the SBRC gastric phase to accurately predict As-RBA in contaminated soils (Juhasz et al., 2014). In this study, there was insignificant difference between As bioaccessibility based on the GP of SBRC assay and As-RBA for soils A and B (Figure 5), suggesting that the gastric phase of SBRC might be suitable to predict As-RBA in contaminated soil.

A different trend was observed for the As-RBA in two soil samples with treated by 1% or 2% nZVI. There was a significant difference between As bioaccessibility based on GP or IP of SBRC assay and As-RBA after being treated with nZVI for 56 days. Based on the variable effect of nZVI on As-RBA and bioaccessibility, the SBRC assay was unsuitable to predict As-RBA in nZVI amended soils. This may be due to the influence of nZVI adsorbed with As increasing As accumulation during *in vivo* assay, while reducing As available fraction during *in vitro* assay. To accurately assess the nZVI human health risk, As-RBA should be considered in concert with secondary evidence provided through fraction and bioaccessibility assessments. In addition, it is necessary to develop suitable *in vitro* assay to predict As-RBA in nZVI-amended soils.

## Conclusion

In this study, two different soils were selected for study. Using the Tessier sequential extraction method, it was found that there was a great difference in the content of As in E1+C2 between the two soils. As bioaccessibility in two kinds of soils treated with nZVI was determined by SBRC method, and As-RBA was determined by mice liver and kidneys model. The *in vitro* simulation results showed that nZVI significantly reduced the bioaccessibility of As in GP and IP from 21.2–79.2% and 13.9–51.6% to 9.85–83.87% and 0.36–9.96%, respectively. Different from As bioaccessibility, nZVI has the possibility of increasing As-RBA from 21.6% to 22.3% and 39.9% with 1% and 2% dosages for soil A. For sample B, the As-RBA was reduced from 73.0% to 55.3% and 68.9% after addition 1% and 2% nZVI for 56 days. The amount of As fraction in E1 and C2 may be critical in influencing the As-RBA compared to the aging soil composition of samples A and B.

Through the analysis of the difference between As bioaccessibility and relative bioavailability, there was no significant difference between As in bioaccessibility in GP and As-RBA in untreated soil A and B, but there was a significant difference between As bioaccessibility in GP and As-RBA in nZVI treated soil A and B. The addition of nZVI reduced the accuracy of SBRC method in predicting As-RBA in soil A. In order to further determine the nZVI remediation risk of arsenic contamination soil, more soil samples with different types, different As concentrations and nZVI concentrations should be included. In addition, it is necessary to develop a new *in vitro* method for better prediction of As-RBA for nZVI-amended soils.

## Data Availability

The original contributions presented in the study are included in the article/Supplementary Material; further inquiries can be directed to the corresponding author.
