# Supplementary material for: Effect of Nanoscale Zero-Valent Iron on Arsenic Bioaccessibility and Bioavailability in Soil
Source: Front Chem. 2022 Jul 22;10:964893. doi: 10.3389/fchem.2022.964893 (PMC9353111; doi:10.3389/fchem.2022.964893)
Supplement: Supplementary file 1 [file DataSheet1.docx]

**Supporting Information**

**Effect of nanometer zero-valent iron on arsenic bioaccessibility and bioavailability in soil**

Shuo Chen^1†^, Lei Han^1,2†^, Qiu Wang^3^, Chenglang Liu^1^, Yuzhen, Liu^1^ and Jie Li^1^*

^1^ College of Geography and Environment, Shandong Normal University, Jinan, China

^2^ Jinan Environmental Research Institute (Jinan Yellow River Basin Ecological Protection Promotion Center), Jinan, China

^3^Jinan Ecological Environment Bureau Licheng Branch Bureau, Jinan, China

*Corresponding authors, College of Geography and Environment, Shandong Normal University, Jinan 250358, China; E-mail: lijie2017@sdnu.edu.cn.

**Table S1 Soil As Concentration, Soil Amount Incorporated into Diet, As Concentration in Diet, Food Consumption for mice, and As Dose Level for 2 Soils Utilized in the Mouse Bioassay.**

| Sample | As in soil (mg kg^-1^) | soil amended into diet (%) | As in soil-amended diet  (mg kg^-1^) | food consumption by each mouse  during 10 d period (g) | As dose level  (µg As g^-1^ bw) |
| --- | --- | --- | --- | --- | --- |
| STD 1 |  |  | 1 | 26.0±4.35 | 0.13±0.02 |
| STD 2 |  |  | 5 | 26.5±4.14 | 0.70±0.09 |
| STD 3 |  |  | 20 | 29.9±4.23 | 3.07±0.49 |
| STD 4 |  |  | 100 | 25.7±2.02 | 13.4±1.50 |
| SA | 129.47 | 3.33 | 4.31 | 43.0±2.77 | 0.82±0.05 |
| SA-1%nZVI-1d | 129.47 | 3.33 | 4.31 | 32.6±3.67 | 0.68±0.06 |
| SA-1%nZVI-56d | 129.47 | 3.33 | 4.31 | 34.9±2.18 | 0.70±0.01 |
| SA-2%nZVI-1d | 129.47 | 3.33 | 4.31 | 34.5±4.83 | 0.71±0.12 |
| SA-2%nZVI-56d | 129.47 | 3.33 | 4.31 | 31.1±1.91 | 0.65±0.03 |
| SB | 121.16 | 3.33 | 4.03 | 27.9±3.67 | 0.55±0.04 |
| SB-1%nZVI-1d | 121.16 | 3.33 | 4.03 | 34.5±2.35 | 0.65±0.04 |
| SB-1%nZVI-56d | 121.16 | 3.33 | 4.03 | 36.7±2.49 | 0.70±0.05 |
| SB-2%nZVI-1d | 121.16 | 3.33 | 4.03 | 30.3±7.50 | 0.58±0.11 |
| SB-2%nZVI-56d | 121.16 | 3.33 | 4.03 | 36.3±2.69 | 0.67±0.07 |

**Table S2 The concentration of free iron was obtained by DCB extraction. The concentration of iron oxide was obtained by oxalic acid-ammonium oxalate extraction method.**

| Sample | Fe from DCB | Fe from Oxalic acid-ammonium oxalate (g kg^-1^) |
| --- | --- | --- |
|  | (g kg^-1^) |  |
| SA | 14.3±0.62 | 2.65±0.08 |
| SA-1% nZVI-3d | 21.6±0.41 | 8.21±0.47 |
| SA-1% nZVI-56d | 22.0±1.50 | 10.5±0.79 |
| SA-2% nZVI-3d | 30.3±1.93 | 17.7±0.29 |
| SA-2% nZVI-56d | 35.8±1.09 | 21.5±0.76 |
| SB | 8.69±0.81 | 8.00±1.02 |
| SB-1% nZVI-3d | 15.6±0.72 | 14.3±1.41 |
| SB-1% nZVI-56d | 17.9±0.78 | 14.8±2.50 |
| SB-2% nZVI-3d | 29.3±0.09 | 24.5±0.79 |
| SB-2% nZVI-56d | 31.3±0.54 | 30.4±0.95 |

**Table S3 Pearson correlation between bioaccessible Fe concentration and As bioaccessibility in nZVI treated soil samples (* indicates that the correlation is significant at level 0.05, and * * means that the correlation is significant at level 0.01)**

|  |  |  | Fe concentration (mg kg^-1^) | | | |
| --- | --- | --- | --- | --- | --- | --- |
|  |  |  | Soil A | | Soil B | |
|  |  |  | GP | IP | GP | IP |
| As bioaccessibility (%) | Soil A | GP | -0.227 | 0.029 |  |  |
|  |  | IP | -0.600^*^ | 0.470 |  |  |
|  | Soil B | GP |  |  | 0.218 | 0.263 |
|  |  | IP |  |  | -0.718^**^ | -0.657^**^ |

**Figure S1 Linear relationship between As concentration in (a) mouse liver, (b) mouse kidneys, (c) both liver and kidneys and As dosed as sodium arsenate.**
